# Incidence and risk factors of delayed postoperative hyponatremia after endoscopic endonasal surgery for Rathke’s cleft cyst: A single-center study

**DOI:** 10.3389/fsurg.2022.953802

**Published:** 2022-07-15

**Authors:** Ao Qian, Jing Zhou, Jiaojiao Yu, Gang Huo, Xiaoshu Wang

**Affiliations:** Department of Neurosurgery, The First Affiliated Hospital of Chongqing Medical University, Chongqing, China

**Keywords:** Hyponatremia, Rathke’s cleft cyst, endoscope, suprasellar, SIADH

## Abstract

**Backgroud:**

Delayed postoperative hyponatremia (DPH) is common for sellar lesions. However, the true prevalence and associated factors of DPH after endoscopic endonasal surgery (EES) for Rathke’s cleft cyst (RCC) have not been studied in a large patient cohort.

**Methods:**

A retrospective analysis was conducted over 6 years at our institution, and patients with RCC treated by EES were enrolled according to our inclusion criteria. Patient demographics, clinical characteristics, images, and surgical procedures were documented. Serum sodium was routinely measured before surgery, on postoperative day 1, and every 2 days thereafter until discharge. For patients with DPH, electrolyte, hematocrit, serum protein levels, and plasma and urinary osmolality were daily measured to explore potential etiology.

**Results:**

Of the 149 eligible patients, 25 (16.8%) developed DPH, which was similar to other sellar lesions, except craniopharyngioma, in the same period in our institution. Significant risk factors suggested by univariate analysis were cyst location, requirement of postoperative hydrocortisone therapy, postoperative meningitis, intraoperative cerebrospinal fluid (CSF) leakage, and subtotal resection (STR) of the cyst wall (all *p* < 0.05). In addition, other supplementary 11 cases of suprasellar RCC with different surgical strategies (aggressive resection) and relevant factors were enrolled into multivariate analysis. Suprasellar location [odds ratio (OR) 8.387, 95% confidence interval (CI) 1.014–69.365, *p* = 0.049], requirement of postoperative hydrocortisone therapy (OR 4.208, 95%CI 1.246–14.209, *p* = 0.021), and intraoperative CSF leakage (OR 6.631, 95%CI 1.728–25.440, *p* = 0.006) were found to be the independent predictors of DPH.

**Conclusion:**

DPH is a common complication after EES for RCC. Suprasellar location, requirement of postoperative hydrocortisone therapy, and intraoperative CSF leakage are the most reliable risk factors. Cortisol deficiency and syndrome of inappropriate antidiuretic hormone (SIADH) are considered as the main etiologies of DPH in RCC. Conservative excision of the cyst wall may reduce DPH occurrence.

## Introduction

Hyponatremia is a common complication following surgery for sellar lesions, as well as the main reason for readmission after the removal of pituitary tumors (1). Data from previous studies indicate that hyponatremia is correlated with prolonged postoperative hospital stay after transsphenoidal surgery, although not obviously increasing mortality (1, 2). However, serious complications may be produced with drastic fluctuation in the level of serum sodium, like central pontine myelinolysis and extrapontine myelinolysis, which caused by rapid correction of hyponatremia (3). Symptomatic hyponatremia generally occurs only if the sodium level drops to 125 mEq/L, and patients may present with headache, fatigue, nausea, and vomiting. Serious neurologic symptoms may develop if inadequate treatment is adopted for progressive hyponatremia, including altered mental status and seizure (4). Hyponatremia in the early postoperative period [before postoperative day (POD) 3] is mostly attributed to improper fluid management (5), while delayed postoperative hyponatremia (DPH) usually results from the syndrome of inappropriate secretion of antidiuretic hormone (SIADH) or hypocortisolemia, rarely from cerebral salt-wasting syndrome (6, 7).

Rathke’s cleft cyst (RCC) is the most common non-neoplastic lesion in the sellar region arising from the remnant of Rathke punch (8). Most RCCs originate in the pars intermedia, between anterior and posterior pituitary lobes, with or without suprasellar extension, while a minority of RCC subtype entirely locates above the diaphragm (9, 10). With the development of the endoscopic technique, endoscopic endonasal surgery (EES) has become the main therapeutic method for RCC, even suprasellar RCC that craniotomy is traditionally required can acquire satisfying outcome by extended variation of EES (11, 12). There is a large volume of published studies describing the role of DPH in pituitary tumors or general sellar lesions (13–16); however, clinical and cystic characteristics that are predictive of DPH in RCC have not been clearly delineated. Therefore, we retrospectively analyze our series of RCCs treated by EES to clarify the incidence and risk factors of DPH in RCC.

## Methods

### Study design

This study has been approved by the Ethics Committee in our institution. All patients undergoing EES for the treatment of RCC at the First Affiliated Hospital of Chongqing Medical University from January 2015 to March 2021 were considered in this study. Peri-operative management and surgery were performed by a same group of senior surgeons. Patients were excluded in this study if they suffered from comorbidities of kidney or adrenal diseases, if they had heart disease of decompensated cardiovascular function, or diabetes mellitus patients with poor blood sugar control. Meanwhile, patients with hyponatremia in the early postoperative period (POD 1 or 2) were also not included. Relevant data, including demographics, clinical characteristics, imaging findings, and surgical procedures, were reviewed. Pituitary function was evaluated by laboratory assessment of adrenocorticotropic hormone, thyroid-related hormones, cortisol level, and other pituitary-related hormones. The variation in the signal intensity of cyst contends on T1- and T2-weighted MRI was recorded. Classification of cyst location (intrasellar, intrasuprasellar, and suprasellar), maximum diameter, and enhancement of cyst wall were evaluated through Gd-enhanced T1-weighted MRI. In addition, other sellar lesions, including pituitary adenoma, tuberculum sellae meningioma, chordoma, and craniopharyngioma, which met the above inclusive criteria, were also collected to compare the incidence of DPH with RCC.

### Protocol for hyponatremia

Hyponatremia was defined as serum sodium concentration <135 mEq/L and rated as three grades: mild (130–134 mEq/L), moderate (125–129 mEq/L), and severe (<125 mEq/L) (4). Our protocol for peri-operative fluid/sodium management is listed as follows:

1. Serum sodium was routinely measured before surgery, on POD1, and every 2 days thereafter until discharge.
2. For patients with preoperative hyponatremia, sodium level should be restored to normal before surgery.
3. Uneventful patients began normal diet on POD 1, and intravenous transfusion was gradually evacuated in 2–3 days.
4. For patients with DPH, therapeutic regimens of water restriction and/or sodium supplementation were applied according to daily monitoring of electrolyte, hematocrit, serum protein levels, and plasma and urinary osmolality until serum sodium levels became normal.
5. The normalization of sodium level should be gradual with precise control (fluctuation < 10 mEq/L per 24 h), and patients were discharged with serum sodium concentration >130 mEq/L and relief of symptoms.
6. For discharged patients, electrolyte and pituitary-related hormones were measured in outpatients on POD 7, 14, and 30, and patients were readmitted if they developed symptomatic DPH or moderate-to-severe hyponatremia.
7. For patients with hypopituitarism, a physiological dose of hormonal replacement was given.
8. For patients with DI (whether preoperative or new onset), during hospitalization, in addition to daily monitoring of serum electrolyte and plasma and urinary osmolality, hourly urine volume was also accurately recorded to ensure that our antidiuretic hormone (ADH) supplementation was precise and individual-based.

### Surgical strategies

All patients with intrasellar or intrasuprasellar RCC were treated by standard EES, and routinely full drainage of cyst content with a partial resection of the cyst wall was implemented. Extended EES (transtuberculum/transplanum surgery) was performed for suprasellar RCC. A moderate strategy of “maximal resection with safety” was adopted in cases with suprasellar RCC; that is, functional preservation of surrounding structures was prioritized, rather than excessive excision of the cyst wall.

### Additional supplementary cases

In our early period of practice, a relatively aggressive strategy attempting to achieve gross total resection (GTR) of the cyst wall was performed on 11 patients with suprasellar RCC. This aggressive surgical strategy was distinct from the majority in our cohort. Considering surgical regimen is indeed an important factor affecting hyponatremia, these patients were only enrolled into multivariate analysis to evaluate the independent factors of DPH but not used to assess the general incidence and univariate analysis.

### Statistical analysis

Variables in univariate analysis were selected based on previous studies (17, 18) and our experience. The variables of “requirement of postoperative hydrocortisone therapy” and “Requirement of postoperative thyroxine therapy” referred to patients who needed hormone replacement due to either newly developed or preoperative hypopituitarism (without postoperative recovery). Univariate analysis was performed using the Student’s *t*-test for continuous variables if normal distribution was satisfied, otherwise, the Mann–Whitney *U*-test was performed. Pearson chi-square or Fisher’s exact test were performed for categorical variables. When exploring the independent predictors of DPH, variables with statistical significance in univariate analysis and the mentioned supplementary cases were enrolled into multivariate analysis *via* binary logistic regression. Furthermore, the variables enrolled into multivariate analysis were also selected to explore the correlations with the grade of hyponatremia by using the Spearman correlation test. All statistical analyses were carried out using SPSS 25.0. For all tests, *p* < 0.05 was considered statistically significant.

## Results

### Demographics

**Figure 1:**
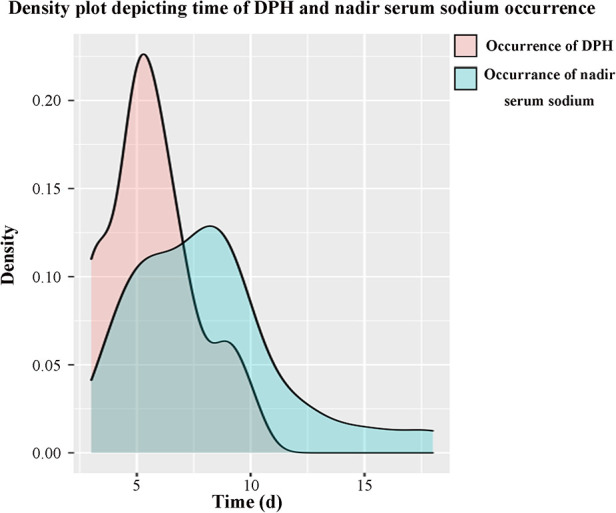
Time distribution of DPH and nadir serum sodium occurrence. The onset of hyponatremia was most common on POD 5, mean POD (5.7 ± 1.9), from POD 3 to 10. The occurrence of nadir sodium was most common on POD 9, mean POD (8.1 ± 3.4), from POD 4 to 18.

**Figure 2:**
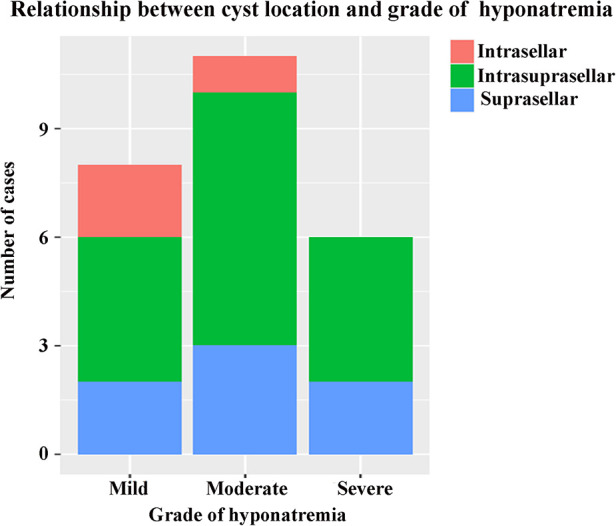
Frequency of cyst location in different grades of hyponatremia.

A total of 149 patients were eligible and enrolled into this study, consisting of 75 (50.3%) females and 74 (49.7%) males, with a mean age of 43.0 ± 14.4 years. Among the cohort, 25 (16.8%) patients developing DPH within 30 days were regarded as the hyponatremia group, and the remaining 124 patients served as the control group. A similar baseline serum sodium was detected before and in the early period after surgery, and there was no significant difference in terms of sex ratio, age, and body mass index (BMI) between these two groups (Table 1). The onset of hyponatremia and occurrence of nadir sodium level were most common on POD 5 and POD 9, ranging from POD 3 to 10 and POD 4 to 18, respectively (Figure 1). The incidence of DPH in different grades is summarized in Table 2 and depicted in Figure 2. Two patients were readmitted because of DPH. Further, a similar incidence of DPH was observed in RCC and other sellar lesions (all *p* > 0.05), except craniopharyngioma (*p* = 0.029) (Table 3).

**Table 1:** Univariate analysis of factors associated with DPH.

Variables	With DPH, *n* (%)	Without DPH, *n* (%)	*p* value
Demographics			
No. of patients	25	124	
Female sex, *n* (%)	13 (52.0)	62 (50.0)	0.855
Mean age, year	44.8 ± 15.4	42.7 ± 14.2	0.504
BMI	23.9 ± 4.9	24.0 ± 3.8	0.927
Baseline of serum sodium (mEq/L)			
Preoperative	140.2 ± 4.7	140.6 ± 4.6	0.907
Early postoperative	138.9 ± 2.7	139.5 ± 3.0	0.473
Cyst locations			<0.001
Intrasellar	3 (12.0)	51 (41.1)	
Intrasuprasellar	15 (60.0)	68 (54.8)	
Suprasellar	7(28.0)	5 (4.0)	
Cyst features			
T1 signal			0.237
Hypointense	13 (52.0)	46 (37.1)	
Isointense	7 (28.0)	56 (45.2)	
Hyperintense	5 (20.0)	22 (17.7)	
T2 signal			0.953
Hypointense	6 (24.0)	35 (28.2)	
Isointense	4 (16.0)	20 (16.1)	
Hyperintense	15 (60.0)	69 (55.6)	
T1 rim enhancement	6 (24.0)	35 (28.2)	0.666
Crystals in cyst	10 (40)	42 (33.9)	0.558
Cyst diameter (mm)	16.2 ± 4.0	15.3 ± 4.2	0.295
Recurrent cyst	0 (0)	4 (3.2)	1.0
Clinical characteristics			
History of hypertension	5 (20.0)	33 (26.6)	0.489
History of diabetes mellitus	5 (20.0)	14 (11.3)	0.320
Preoperative DI	5 (20.0)	17 (13.7)	0.535
Preoperative hypopituitarism	8 (32.0)	40 (32.3)	1.0
Requirement of postoperative hydrocortisone therapy^a^	10 (40.0)	16 (12.9)	0.003
Requirement of postoperative thyroxine therapy^a^	4 (16.0)	14 (11.3)	0.507
Preoperative hyponatremia	4 (16.0)	9 (7.3)	0.234
Postoperative CSF leakage	1 (4.0)	2 (1.6)	0.426
Postoperative meningitis	5 (20.0)	2 (1.6)	0.002
New-onset DI	4 (16.0)	9 (7.3)	0.234
Intraoperative characteristics			
Intraoperative CSF leakage	16 (64.0)	14 (11.3)	<0.001
STR of the cyst wall^b^	3 (12.0)	1 (0.8)	0.015

^a^ *Replacement therapy at discharge.*

^b^ *Resection degree >90%.*

*RCC, Rathke’s Cleft Cyst; DPH, delayed postoperative hyponatremia; BMI, body mass index; DI, diabetes insipidus; CSF, cerebrospinal fluid; STR, subtotal resection.*

**Table 2:** Incidence of DPH in different grades.

Grade, *n* (%)	Total number	Symptomatic	Intrasellar	Intrasuprasellar	Suprasellar
Mild	8 (5.4)	3 (2.0)	2 (1.3)	4 (2.7)	2 (1.3)
Moderate	11 (7.4)	6 (4.0)	1 (0.7)	7 (4.7)	3 (2.0)
Severe	6 (4.0)	3 (2.0)	0	4 (2.7)	2 (1.3)

**Table 3:** Comparison of the DPH rate in RCC and other sellar lesions.

Lesions	Total, *n*	Cases with DPH, *n* (%)	*p* Value (compared with RCC)
RCC	149	25 (16.8)	–
Pituitary adenoma	874	173 (19.8)	0.389
Craniopharyngioma	142	47 (33.1)	0.001
TSM	24	4 (16.7)	1.0
Chordoma	27	5 (18.5)	0.785

*DPH, delayed postoperative hyponatremia; RCC, Rathke’s Cleft Cyst; TSM, tuberculum sellae meningioma.*

### DPH and characteristics of RCC

Among the entire cohort, 54 (36.2%) intrasellar, 83 (55.7%) intrasuprasellar, and 12 (8.1%) suprasellar RCCs were identified. Compared with the former two types, suprasellar RCC had a significantly higher incidence of DPH (*p* < 0.001). However, other imaging characteristics, such as cyst diameter, enhancement of cyst wall, and variation in the signal intensity of cyst contends on T1- and T2-weighted MRI, were not significantly associated with DPH.

### Clinical and surgical features

Partial resection of the cyst wall with full content drainage was performed in all patients with intrasellar and intrasuprasellar RCC and in eight patients with suprasellar RCC. However, a subtotal resection of the cyst wall (STR, a resection degree of more than 90%) was used in the remaining four suprasellar patients in whom the cyst wall could be safely dissected. Comparisons of the peri-operative pituitary function and complications demonstrated that postoperative meningitis and the requirement of postoperative hydrocortisone therapy were significantly associated with the incidence of DPH (both *p* < 0.05). Furthermore, intraoperative cerebrospinal fluid (CSF) leakage and STR of cyst wall also significantly indicated the rate of DPH (both *p* < 0.05).

### Characteristics of supplementary cases

Among the 11 patients with suprasellar RCC experiencing aggressive resection, 10 (90.9%) developed DPH. In light of the fact that distinct surgical strategies were used in our total suprasellar cases, we attempted to investigate the effect of this difference on suprasellar RCC with patients possessing at least 1 year follow-up. As summarized in Table 4, we noticed an obviously higher incidence of DPH and hypopituitarism in patients with aggressive resection of the cyst wall, compared with those experiencing moderate cystic excision (90.9% vs. 58.3% and 36.4% vs. 0%, respectively).

**Table 4:** Effect of different resection strategies on suprasellar RCC.

Variables, *n* (%)	Aggressive resection	Maximal safe resection
No. of patients	11	12
DPH	10 (90.9)	7 (58.3)
Hypopituitarism^a^	4 (36.4)	0
Diabetes insipidus		
Permanent	2 (18.2)	0
Transient	1 (9.1)	3 (25)
Recurrence	2 (18.2)	2 (16.7)

^a^ *Pituitary function at the last follow-up.*

*DPH, delayed postoperative hyponatremia.*

### Factors associated with DPH for RCC

**Figure 3:**
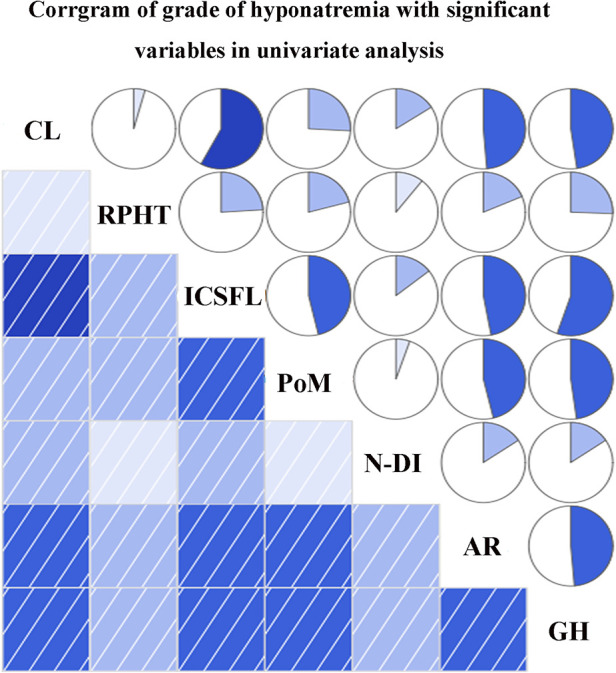
Correlations between grade of hyponatremia and variables enrolled into multivariate analysis. CL, cyst location; RPHT, requirement of postoperative hydrocortisone therapy; ICSFL, intraoperative cerebrospinal fluid leakage; PoM, postoperative meningitis; N-DI, new-onset diabetes insipidus; AR, aggressive resection; GH, grade of hyponatremia.

The variables identified had significant differences in univariate analysis between the hyponatremia group and the control group, and these related to cyst location, requirement of postoperative hydrocortisone therapy, postoperative meningitis, intraoperative CSF leakage, and STR of the cyst wall. In addition, although significance was not found, we considered that new-onset cerebrospinal fluid (DI) (whether permanent or transient) was also a vital factor associated with sodium disorder and enrolled into multivariate analysis. Multivariate binary logistic regression demonstrated suprasellar cyst location (OR 8.387, 95%CI 1.014–69.365, *p* = 0.049), hydrocortisone therapy on discharge [odds ratio (OR) 4.208, 95% confidence interval (CI) 1.246–14.209, *p* = 0.021], and intraoperative CSF leakage (OR 6.631, 95%CI 1.728–25.440, *p* = 0.006) as the independent predictors of DPH in RCC (Table 5). We further explored the relationship between the grade of hyponatremia and those variables with statistical significance in univariate analysis, finding that the degree of hyponatremia had a relatively high correlation with cyst location, intraoperative CSF leakage, postoperative meningitis, and aggressive resection of the cyst wall (*r*_s_ > 0.4, all *p* < 0.001), while weakly associated with the requirement of postoperative hydrocortisone therapy and new-onset DI (*r*_s_ < 0.4, both *p* < 0.05) (Figure 3).

**Table 5:** Multivariate analysis identifying independent predictors of DPH.

Parameter	OR	95%CI	*p* Value
Suprasellar location	8.387	1.014–69.365	0.049
Requirement of postoperative hydrocortisone therapy	4.208	1.246–14.209	0.021
Intraoperative CSF leakage	6.631	1.728–25.440	0.006

*CSF, cerebrospinal fluid.*

## Discussion

Hyponatremia is not rare in patients with sellar lesion after EES. Although extensive research has been carried out on DPH in sellar lesions, the proportion of RCC involved was definitely low (19–21). Kunal et al. (22) studied 367 cases of sellar lesions with a 15% incidence of DPH, but RCC was accounted for only 10% of the total. Namath et al. (6) reported a series of 373 lesions in the sellar and parasellar regions with DPH in 15% as well; however, only 10 RCCs were enrolled in their cohort. In our study, comparisons of the DPH rate in RCC and other sellar lesions demonstrated similar incidence in RCC and pituitary adenoma, underlining the importance of perceiving this complication in RCC. At present, in reviewing the literature, no detailed data were found on the incidence and risk factors of DPH in RCC, and to our knowledge, the current study is the first to describe the characteristics of hyponatremia in the case series of RCC.

### Factors and etiology of DPH

The association of old age, low BMI, female sex, and the size of tumor with DPH in sellar lesions has been proposed previously (6, 14, 22–24). In contrast to our earlier findings, however, no obvious correlation between DPH and demographics was detected in our cohort. Compared with pituitary tumors, rim enhancement of the cyst wall and various signals of cystic content on T1- and T2-weighted MRI were unique in RCC. However, the observational connection between imaging characteristics and DPH was not significant. The current study found that patients requiring postoperative hydrocortisone therapy were more prone to develop DPH. A possible explanation for this result might be that rather than a paradoxical effect of the treatment itself, patients with postoperative hypocortisolemia possessed a more erratic fluctuation of serum cortisone, even with a physiological dose of hydrocortisone replacement, and were more likely to have a relatively deficient cortisone level, particularly under stress conditions, such as meningitis, making them more susceptible to hyponatremia. Thus, for patients needing hydrocortisone therapy after surgery, more attention should be paid to the electrolyte status when ensuring adequate hormone replacement.

Besides hypocortisolemia and hypothyroidism, SIADH was regarded as another common etiology of hyponatremia after surgery for pituitary lesions (16, 25, 26). This is mostly due to the unregulated release of arginine vasopressin caused by intraoperative neuro-hypophyseal trauma. In the hyponatremia group, no hypovolemia was detected by the measurements of hematocrit and serum protein levels, and decreased serum osmolality with normal or elevated urine osmolality was found in all patients consistent with SIADH (26, 27). Even after excluding patients with postoperative hypopituitarism, SIADH was also considered in 60% (15/25) of the patients. Therefore, it is suggested that SIADH could be a major etiology, if not the only one, causing DPH in the RCC of our cohort.

### Relationship between DPH and RCC location

Different from the two subtypes of intrasellar origin (intrasellar and intrasuprasellar RCC), suprasellar RCC originates from the pars tuberalis of the pituitary gland above the diaphragm, which is more closely related to structures in the suprasellar region (11). Although uncommon, suprasellar RCC serves as a special type of this lesion. Previous studies have reported a higher incidence of recurrence and a lower rate of symptomatic relief in suprasellar RCC after surgery (11, 28). Further, we found that RCC in a purely suprasellar location was also an independent predictor of DPH. While the suprasellar subtype constituted a small proportion of the cohort (12 of 149), and similar incidence of DPH was found in current and previous studies (22, 29), suprasellar RCC accounted for about a third of the total (7/25). Moreover, suprasellar RCC constituted a proportion of more than 70% (5 of 7) in patients with moderate to severe degrees of hyponatremia. Variables, significantly associated with DPH in univariate analysis, appeared to be mostly influenced by the suprasellar location, such as intraoperative CSF leakage, postoperative meningitis, and the resection degree of the cyst wall. Spearman’s correlation test, which was done after enrolling supplementary cases, also indicated the same outcomes. Although statistical significance existed in analyses, there was a strong correlation between these factors and the suprasellar location in clinical practice. In our cohort, extended EES was performed for all patients with suprasellar RCC, requiring dissecting the dura at the tuberculum sellae and directly reaching the suprasellar cistern. Therefore, considerably large skull base defects with intraoperative high-flow CSF leakage existed in these patients, which obviously increased the risk of postoperative meningitis. Ultimately, in addition to cortisol disorder, suprasellar location may be another major factor of DPH in RCC.

We opine that, similar to other analogical studies, SIADH is also the main etiology of DPH in RCC. However, unlike nosogenesis in pituitary tumor, mostly caused by intraoperative damage of neurohypophysis (30), the disorder of ADH release after surgery for RCC is mainly due to its influence on the pituitary stalk. First, successful decompression of RCC main acutely recover the chronically deformed pituitary stalk accompanying with excessive release of ADH. Second, the damage or manipulation on stalk, especially during the surgical procedure of suprasellar RCC, increased the early release of ADH (5). In the hyponatremia group with enrollment of supplementary cases, six of the seven patients developing postoperative DI had suprasellar RCC, with three patients presenting with a typical triphasic response (polyuria–hyponatremia–polyuria) (9) and the remaining three patients presenting with a biphasic response (polyuria–hyponatremia) (4). On the contrary, given the farthest distance from the pituitary stalk, only 3 of 54 patients with a purely intrasellar RCC experienced DPH. Variables must be interpreted with caution, especially those that were significant in univariate or even multivariate analysis, such as intraoperative CSF leakage, because most factors were inherently influenced by the suprasellar location. Thus, predictors obtained in this study were only based on statistical inference, and the results should be interpreted according to clinical practice. In other words, SIADH in RCC may be ultimately determined by cyst location.

### Cyst resection

The surgical treatment of RCC has improved with advances in technique and a better understanding of the natural characteristics of this lesion, while the extent of cystic resection has long been controversial. Previous studies have mostly focused on the correlation between recurrence or postoperative pituitary function and the extent of cystic resection, suggesting that the GTR of the cyst wall appeared to reduce recurrence, but indeed elevated the risk of postoperative DI and hypopituitarism (11, 31, 32). In the current study, a new strategy of maximal safe resection for the cyst wall was provided from the perspective of avoiding DPH. Unfortunately, it is inaccurate to investigate the true relationship between cyst resection and DPH in our patients with intrasellar or intrasuprasellar RCC further, since only a full drainage of content with partial cyst resection was performed. However, the incidence of DPH in these two types of RCC was 13.1%, which was also at a relatively low level compared with that in previous studies (22, 29). An aggressive strategy was attempted for dealing with suprasellar RCC in our early practice on 11 patients, compared with maximal safe resection for late 12 cases. A comparison of the results between these two cyst resection strategies indicated that a relatively conservative cystic excision may achieve better functional protection without an obviously higher advance in recurrence. However, statistical analysis was dropped, given the limited size of the cohort. Unlike pituitary tumors requiring complete resection as far as possible to reduce the recurrence, which may increase the risk of neurohypophysis injury and hyponatremia, it was unclear whether an aggressive resection of the cyst wall was more beneficial for RCC, especially the suprasellar type, at present. The current study identified the comparatively low rate of DPH for intrasellar or intrasuprasellar RCCs with a complete drainage of cyst content and a partial resection of the cyst wall, while there is a higher risk of hyponatremia for suprasellar patients experiencing aggressive cystic resection. The strategy of maximal safe resection better preserved the stalk or pituitary tissue distributed on the cyst wall without obviously increased recurrence within current observation, reducing the risk of postoperative hypopituitarism and DPH. Therefore, we considered that, as a benign lesion with great prognosis, priority should be given for the protection of pituitary stalk and hypothalamus during the surgical management of RCC, particularly the suprasellar type, rather than opting for the riskier radical resection of the cyst wall. Even for partial suprasellar RCC with loose adhesion to surrounding structures, the cyst wall could be safely dissected, and postoperative serum electrolyte should also be closely monitored.

Certainly, some limitations also existed in our study. First, potential bias may be introduced for the retrospective analysis and the limited size of the cohort in a single institution. Second, measurement of serum sodium at discrete time points may miss the possible transient hyponatremia. Third, other potential variables that may be associated with DPH have not been thoroughly dissected in our study. Finally, some results were obtained based on statistical inference, and interpretation should be combined with clinical practice.

## Conclusion

DPH is a common and non-negligible complication after EES for RCC, and it is necessary to strengthen the monitoring of postoperative electrolyte. Attention should be paid to patients with suprasellar RCC, intraoperative CSF leakage, and postoperative hydrocortisone therapy. Cortisol deficiency and SIADH are the main etiologies of DPH in RCC, and the manipulation of pituitary stalk may be the main cause of SIADH. For surgical treatment of suprasellar RCC, priority should be given to the protection of pituitary stalk and hypothalamus, rather than an aggressive resection of the cyst wall. A thorough preoperative evaluation, circumspect surgical manipulation, and a meticulous postoperative scrutiny of serum sodium should be performed for patients with high risk.

## Data Availability

The raw data supporting the conclusions of this article will be made available by the authors, without undue reservation.
